# Self-Diffusion of Star and Linear Polyelectrolytes in Salt-Free and Salt Solutions

**DOI:** 10.1021/acs.macromol.4c01374

**Published:** 2024-12-27

**Authors:** Aliaksei Aliakseyeu, Erica Truong, Yan-Yan Hu, Ryan Sayko, Andrey V. Dobrynin, Svetlana A. Sukhishvili

**Affiliations:** 1 Department of Materials Science & Engineering, Texas A&M University, College Station, Texas 77840, United States; 2 Department of Chemical Engineering, Texas A&M University, College Station, Texas 77840, United States; 3 Department of Chemistry and Biochemistry, Florida State University, Tallahassee, Florida 32306, United States; 4 Center of Interdisciplinary Magnetic Resonance, the National High Magnetic Field Laboratory, 1800 East Paul Dirac Drive, Tallahassee, Florida 32310, United States; 5 Department of Chemistry, University of North Carolina at Chapel Hill, Chapel Hill, North Carolina 27529, United States

## Abstract

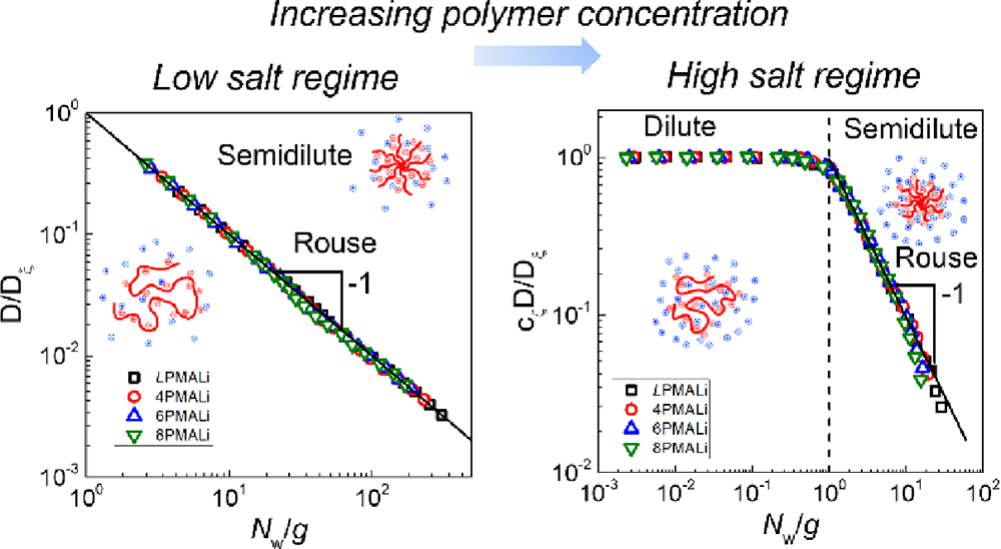

This work explored solution properties of linear and star poly(methacrylic acids) with four, six, and eight arms (*L*PMAA, 4PMAA, PMAA, and 8PMAA, respectively) of matched molecular weights in a wide range of pH, salt, and polymer concentrations. Experimental measurements of self-diffusion were performed by fluorescence correlation spectroscopy (FCS), and the results were interpreted using the scaling theory of polyelectrolyte solutions. While all PMAAs were pH sensitive and showed an increase in hydrodynamic radius (*R*_h_) with pH in the dilute regime, the *R*_h_ of star polymers (measured at basic pH values) was significantly smaller for the star polyacids due to their more compact structure. Fully ionized star PMAAs were also found to be less sensitive to changes in salt concentration and type of the counterion compared to linear PMAA. While *R*_h_ of fully ionized linear PMAA decreased in the series Li^+^ > Na^+^ > K^+^ > Cs^+^ in agreement with the Hofmeister series, *R*_h_ of star PMAAs was virtually independent of type of the counterion for eight-arm PMAA. However, molecular architecture strongly affected interactions of counterions with PMAAs. In particular, ^7^Li NMR revealed that the spin–lattice relaxation time *T*_1_ of Li^+^ ions in low-salt solutions of eight-arm PMAA was ∼2-fold smaller than that in the solution of linear PMAA, suggesting slower Li^+^-ion dynamics within star polymers. An increase in concentration of monovalent chloride salts, *c*_s_, above that of the PMAA monomer unit concentration (*c*_m_) resulted in shrinking of both linear and star molecules, with the hydrodynamic size *R*_h_ scaling as *R*_h_ ∝ *c*_s_^–0.11±0.01^. Self-diffusion of linear and star polyelectrolytes was then studied in a wide range of polyelectrolyte concentrations (10^–3^ mol/L < *c*_m_ < 0.5 mol/L) in low-salt (<10^–4^ mol/L of added salt) and high-salt (1 mol/L) solutions. In both the low-salt and high-salt regimes, diffusion coefficient *D* was lower for PMAAs with a larger number of arms at a fixed *c*_m_. In addition, in both cases, *D* plateaued at low polymer concentrations and decreased at higher polymer concentrations. However, while in the high-salt conditions, the concentration dependence of *D* reflected transitions between the dilute to semidilute solution regimes as expected for neutral chains in good and theta solvents, analysis of the diffusion data in the low-salt conditions using the scaling theory revealed a different origin of the concentration dependence of *D*. Specifically, in the low-salt solutions, both linear and star PMAAs exhibited unentangled (Rouse-like) dynamics in the entire range of polyelectrolyte concentrations.

## Introduction

The distinctive molecular architecture of star polymers^1^ determines their unique properties in solutions^2^ and melts.^3,4^ In the case of neutral stars, the presence of a central branching point leads to redistribution of net polymer mass between arms, resulting in a more compact size of a star in comparison with a size of chemically identical linear chains of equal molecular weights.^2,5−7^ The star size is determined by optimization of the excluded volume interactions and arms’ conformational entropy.^5^ In the case of polyelectrolyte stars, a balance of the osmotic pressure generated by counterions and salt ions and conformational entropy of the arms determines the star size.^8−18^ In salt-free solutions, counterion localization inside the polyelectrolyte stars results in strong arm stretching, which is also directly related to the fraction of ionized groups and the number of arms.^19,20^ The unique feature of such polyelectrolyte star solutions is a broadening of the crossover to semidilute solution regime where stars shrink in size but do not yet interpenetrate.^19^ At high salt concentrations, the electrostatic interactions between ionized groups are reduced to effective short-range interactions with a salt-dependent second virial coefficient. In this high salt concentration regime, polyelectrolyte stars behave as their neutral counterparts.^19^

Solution properties of polyelectrolyte stars were studied by the light, X-ray, and/or neutron scattering and viscosity measurements and concerned interpretation of the “polyelectrolyte” scattering peak emerging as a result of interpolyelectrolyte electrostatic interactions, the effect of molecular architecture on the polyelectrolyte rigidity, and the occurrence of the “abnormal” polyelectrolyte peak in low-salt solutions.^21−30^ These studies revealed, for example, a higher persistence length of polyelectrolyte stars compared to their linear counterparts^22,23^ due to different molecular connectivity and immobilization of arms by the star branching point and due to counterion concentration enhancement within the star volume.^25,28^

However, despite all of these efforts, the dynamics of polyelectrolyte stars in solutions is still poorly understood. This work aims to fill this gap by exploring the self-diffusion of weak polyelectrolytes of different branching ranging from linear chains to eight-arm stars at different polymer and salt concentrations. Using the fluorescent correlation spectroscopy (FCS),^31^ we first compare self-diffusion of stars and linear poly(methacrylic acids) (PMAAs*) in extremely dilute solutions to highlight the effect of branching on hydrodynamic radius as a function of the solution pH and added salt concentration. This is followed by investigation of the self-diffusion of linear and star polyelectrolytes in dilute and semidilute solution regimes at different salt concentrations and salt counterions (Li^+^, Na^+^, K^+^, and Cs^+^).

## Materials and Methods

### Materials

Lithium hydroxide, lithium chloride, sodium chloride, potassium chloride, and cesium chloride were purchased from Thermo Scientific and used as received. Tris(hydroxymethyl)aminomethane (Tris), dimethyl sulfoxide (DMSO), sodium phosphate dibasic, sodium hydroxide, and potassium hydroxide were purchased from Sigma-Aldrich and used as received. Water used in this study was purified using a Millipore Milli-Q system.

Linear (*M*_w_ = 59.8 kDa, *Đ* < 1.1), four-arm (*M*_w_ = 65.2 kDa, *Đ* < 1.1), six-arm (*M*_w_ = 66.7 kDa, *Đ* < 1.2), and eight-arm (*M*_w_ = 65.9 kDa, *Đ* < 1.2) poly(methacrylic acids) were synthesized, characterized, and labeled with Alexa 488 using a protocol described in our previous publications.^31,32^ These polymers, abbreviated as *L*PMAA, 4PMAA, 6PMAA, and 8PMAA for unlabeled polymers and *L*PMAA*, 4PMAA*, 6PMAA*, and 8PMAA* for fluorescently labeled polymers, respectively, had similar molecular weights but different numbers of arms. The degrees of polymerization of *L*PMAA were 695, and 4PMAA, 6PMAA, and 8PMAA star polymers contained four, six, and eight arms composed of 189, 129, and 95 monomer units, respectively. The contour length of the *L*PMAA linear polymer was 174 nm, while the average contour length of the arms of 4PMAA, 6PMAA, and 8PMAA star polymers was 47, 32, and 24 nm, respectively.

### Methods

#### FCS

FCS experiments were performed using a custom-made FCS setup and glass cells that were described in our previous publications.^31,32^ Prior to FCS experiments, the width and the height of the laser beam were calibrated with Alexa 488 dye with the known diffusion coefficient of 440 μm^2^/s. All solutions were left for 5 min for equilibration prior to measurements. The data was collected for 3 min upon continuous exposure to a 4 mW laser excitation. All measurements were performed at room temperature (20 °C).

#### Preparation of Solutions for FCS Measurements of Polymer Diffusion

FCS experiments employed fluorescently labeled polymers with a general abbreviation PMAA*, i.e., *L*PMAA*, 4PMAA*, 6PMAA*, and 8PMAA*, which contained one Alexa 488 label per 790, 1060, 830, and 1030 polymer units, respectively. These polymers were used to either prepare solutions of individually dissolved fluorescent polymers for studies in the dilute polymer concentration regime or mixed with unlabeled PMAA for studies of self-diffusion in a wide range of polymer concentrations 10^–3^ mol/L < *c*_m_ < 0.5 mol/L, where *c*_m_ is the polymer concentration in repeat units.

For FCS studies in dilute polymer solutions, the stock solutions of fluorescently labeled PMAA* were first prepared at a 0.1 mg/mL (*c*_m_ ∼ 10^–3^ mol/L) concentration (corresponding to ∼10^–6^ mol/L Alexa-488-labeled units) using PMAA* powders and DI water. Before measurements, these stock solutions were diluted with either 0.01 mol/L sodium phosphate or 10^–5^ mol/L Tris buffer solutions to achieve the desired pH values. For studies of the effect of salt on polymer diffusion, PMAA* solutions additionally contained LiCl, NaCl, KCl, or CsCl salts.

For studies in a wide range of polymer concentrations, two types of stock solutions were used, i.e., PMAA* stock solutions described above and stock solutions of unlabeled PMAAs. These stock solutions were used to prepare mixed solutions of labeled and unlabeled polymers for FCS measurements. The mixing procedure was required for keeping the concentration of the fluorescent species at a very low level (<10^–9^ mol/L) to ensure good quality of the autocorrelation function. The stock solutions of unlabeled PMAAs were prepared by dissolving 10 mg of PMAA powders in 90 μL of 10^–5^ mol/L Tris buffer, followed by conversion of the protonated form of the polyacids to their salt using different monovalent cations. To that end, LiOH, NaOH, or KOH was added in the equimolar amount of PMAA to units (*c*_m_ ∼ 1.2 mol/L) to create the final concentration of 1.29 mol/L. In these solutions, all cations (Li^+^, Na^+^, or K^+^) were present as counterions. This procedure yielded *c*_m_ ∼ 1.2 mol/L solutions of PMALi, PMANa, or PMAK. The solutions were incubated at ambient temperature overnight to complete polymer dissolution and then used for preparation of solutions for measurements at low-salt concentration conditions in a wide range of polymer concentrations. To that end, 50 μL of unlabeled PMAA stock solutions (*c*_m_ ∼ 1.2 mol/L) were first mixed with 5 μL of PMAA* stock solutions (*c*_m_ ∼ 10^–3^ mol/L), and 45 μL of 10^–5^ mol/L Tris buffer at pH 9 was added. The concentrated PMAA–PMAA* mixtures were placed in custom-made glass cells for FCS measurements. After measurements, the mixed solutions were gradually diluted with 10^–5^ mol/L Tris buffer at pH 9, while small amounts of the stock solution of labeled PMAA* were added to keep the constant level of concentration of the fluorescently labeled polymer units.

For measurements in a wide range of polymer concentrations but in high-salt conditions (1 mol/L LiCl), solutions were prepared in a similar manner, except that the stock solutions of unlabeled PMAAs were at *c*_m_ ∼ 1.76 mol/L (compared to the *c*_m_ ∼ 1.2 mol/L used for the low-salt studies). In addition, both unlabeled and labeled solutions contained 1 mol/L LiCl and 10^–5^ mol/L Tris buffer.

#### ^7^Li NMR Spectroscopy

^7^Li NMR experiments were acquired at room temperature (∼23 °C) and static conditions on a Bruker Avance I spectrometer at a field of 7.05 T with the ^7^Li Larmor frequency of 116.64 MHz. One-dimensional spectra were collected using a single pulse sequence. The T_1_ spin–lattice relaxation times were determined using the inversion–recovery approach . The π/2 pulse length was 3.08 μs. The recycling delay was 5 s. The ^7^Li chemical shift was referenced to 1 mol/L LiCl at 0 ppm. NMR spectra were processed and analyzed by using TopSpin 4.1.4.

#### Preparation of Solutions for ^7^Li NMR Measurements

For ^7^Li NMR measurements, 5.4 mg of PMAA was dissolved in 2.5 mL of 0.25 mol/L LiOH (for low-salt regime measurements) or 0.25 mol/L LiOH in 1 mol/L LiCl (for high-salt regime measurements).

## Results and Discussion

### Dilute Solutions: Effect of pH on Hydrodynamic Size of Linear and Star PMAAs

Diffusion of linear and star polyelectrolytes was explored by using the FCS technique, which can be applied in extremely dilute polymer solutions.^31^ In addition, FCS can be used in an unprecedently wide range of polymer concentrations if a small amount of fluorescent tracer molecules is mixed with nonfluorescent polymer chains.^31,33^ This technique was first applied to studies of the effect of solution pH on the hydrodynamic radius of linear and star PMAAs of matched molecular weight in extremely dilute polymer solutions. For FCS analysis of diffusion of monodisperse species, the diffusion coefficient is calculated from the following equation:
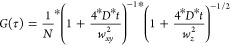
1where  is an autocorrelation function, *D* is a self-diffusion coefficient, *N* is the average number of fluorescent species in the sample volume, and *w*_*xy*_ and *w*_*z*_ are radii of the excitation volume in the *xy*-plane and *z*-directions, respectively.^31,34^ Subsequently, the hydrodynamic radius (*R*_h_) was calculated as follows:
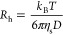
2where *k*_B_ is the Boltzmann constant, *T* is the temperature, and η_s_ is the viscosity of a solvent. Note that the viscosity of a solvent can change as a function of temperature and salt^35^ or cosolvent^36^ concentration, and it has been adjusted appropriately.

**Figure 1:**
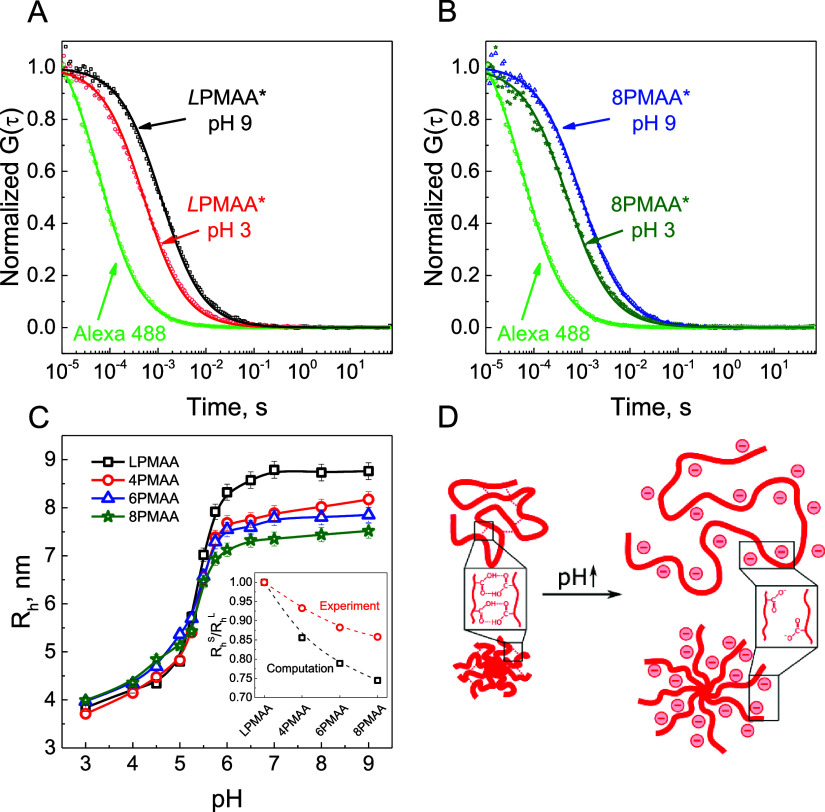
Normalized autocorrelation functions for diffusion of *L*PMAA* (A) and 8PMAA* (B) at pH 3 and 9 shown along with diffusion of free Alexa 488 at pH 9. The data were fitted with the single-component 3D diffusion (eq 1). (C) Effect of pH on hydrodynamic radius of *L*PMAA (squares), 4PMAA (circles), 6PMAA (triangles), and 8PMAA (stars). The polymer concentration, *c*_m_, presented in moles of the repeat polymer units per liter, was *c*_m_ = 10^–5^ mol/L in all solutions. The inset in (C) compares the experimental ratios of *R*_h_s for star and linear PMAAs with the computational simulation for fully charged polyelectrolytes.^2^ (D) Schematic representation of the effect of pH on conformation of linear and star PMAAs. All experiments were performed in 0.01 mol/L sodium phosphate buffer, which additionally contained 0.5 vol % DMSO to prevent aggregation of PMAA at low pH values.

For FCS experiments, linear, four-arm, six-arm, and eight-arm star poly(methacrylic acids) with matched molecular weights (*M*_w_ ≈ 60–66 kDa and *Đ* < 1.2) were covalently modified with Alexa 488 fluorophore, as described in our previous publication.^32^ The synthesized *L*PMAA*, 4PMAA*, 6PMAA*, and 8PMAA* contained one label per 790, 1060, 830, and 1030 polymer units, respectively, as determined by UV–vis spectroscopy.^32^Figure 1A,B shows the normalized autocorrelation functions for the fluorescently tagged *L*PMAA* and 8PMAA* polymers in solutions at different pHs. The characteristic diffusion times (τ) of linear and star PMAA were significantly longer than those observed with free Alexa 488 label (τ = 0.08, 0.96, and 1.15 ms for Alexa 488, *L*PMAA*, and 8PMAA*, respectively, at pH 9), reflecting slower diffusion of polymer molecules, as well as successful polymer labeling and purification of fluorescently labeled PMAAs. The additional control experiment with the equimolar mixture of free Alexa 488 and *L*PMAA chains (Figure S1) illustrates that *G*(τ) for the mixture cannot be successfully fitted with eq 1 for a single species and thus further confirms the absence of unattached fluorescent labels in the experiments shown in Figure 1.

Figure 1C shows that the hydrodynamic radius of both linear and star polymers increased with solution pH, reflecting the rising PMAA ionization. In the range of high solution pH, a clear decrease in *R*_h_ of star polyelectrolytes with an increase in number of arms was observed, in agreement with a theoretical prediction of higher compactness of star polymers.^5^ The experimental ratio of *R*_h_ of star and linear PMAAs (inset in Figure 1C) had a weaker dependence on number of arms than predicted in the computer simulations for linear and star polyelectrolytes,^2^ likely due to higher molecular weights (∼700 monomer units per polymer chain of PMAAs) used in this study as compared to those employed in the computational analysis (∼40 units per polymer chain).

Note that at low pH values, the hydrodynamic sizes of star and linear PMAAs were similar. We hypothesized that this was due to the emergence of intermolecular hydrogen bonding between protonated carboxylic groups that was also found to strongly impact salt response of polycarboxylic acid-containing polyelectrolyte assemblies at low pH.^37,38^ To avoid molecular aggregation at low pH, a small amount (0.5 vol %) of DMSO, a well-known hydrogen donor that breaks hydrogen bonds via a competitive hydrogen bonding with protonated carboxylic groups,^39,40^ was added to all the solutions studied in Figure 1. To directly confirm the contribution of hydrogen bonding to the low pH values of *R*_h_, separated experiments were conducted at a much high DMSO content (95 vol % in water) (DMSO), which demonstrated recovery of the dependence of *R*_h_ on polymer branching (Figure S2). Note that all further experiments were performed at pH 9 where the polyacids were fully ionized and readily dissolved in DMSO-free aqueous solutions.

### Dilute Solutions: Effect of Salt Ions on Hydrodynamic Size of Linear and Star PMAAs

**Figure 2:**
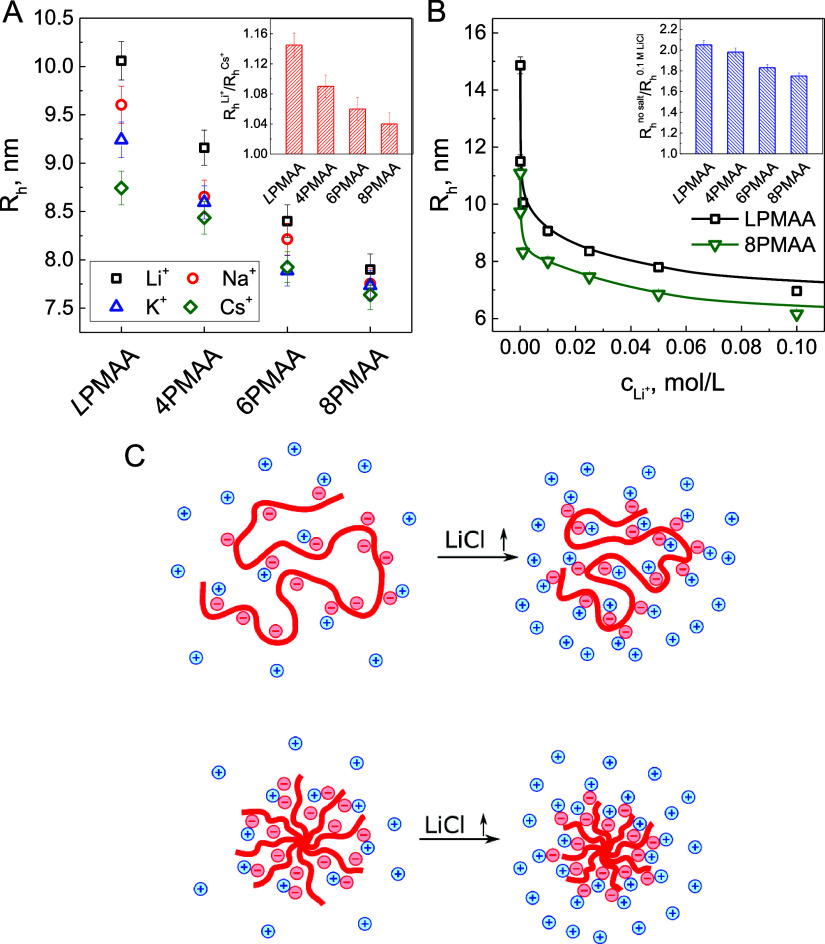
(A) The effect of the type of counterion on hydrodynamic radius of linear and star PMAAs was studied in 0.001 mol/L solutions of LiCl (squares), NaCl (circles), KCl (triangles), and CsCl (diamonds). The inset shows the ratio of polymer *R*_h_s in LiCl and CsCl. (B) Effect of the LiCl concentration on *R*_h_ for *L*PMAA (squares) and 8PMAA (triangles). Th inset shows the ratio of linear and star polyacids *R*_h_s at low-salt solutions (no salt added) to those *R*_h_s in 0.1 mol/L LiCl solutions. All solutions contained *c*_m_ = 10^–5^ mol/L PMAA in 1 × 10^–5^ mol/L Tris buffer at pH 9. (C) Schematic representation of the effect of salt on the conformation of linear and star polyelectrolytes.

Figure 2A shows the effect of the type of cation on the hydrodynamic radii of fully ionized linear and star PMAAs. *R*_h_ of all the polymers measured in dilute solutions in the presence of low concentrations (10^–3^ mol/L) of LiCl, NaCl, KCl, and CsCl. A decrease in *R*_h_ is observed in the following series: Li^+^ > Na^+^ > K^+^ > Cs^+^ is consistent with the Hofmeister series for the monovalent cations and suggests an increasing ability of the cation in the series to screen ionic charges of the polyacids.^41−45^ This effect is related to a larger ionic radius and a smaller hydrodynamic radius of Cs^+^ as compared to Li^+^ ions (1.7 Å vs 1.19 and 0.69 Å vs 2.38 Å for nonhydrated and hydrated Cs^+^ and Li^+^ ions, respectively),^46^ reflecting favorable solvation of lithium ions compared to other alkali cations.^47^ A similar effect was shown for hydrogels of poly(acrylic acid), which demonstrated a higher affinity for Cs^+^ as compared to Li^+^ ions.^48^ The inset of Figure 2A shows that the effect of the cation type was stronger for linear polymers compared to star polymers due to a more compact structure and lower compressibility of the star polymers.

Figure 2B illustrates the effect of salt concentration on *R*_h_ of linear and star PMAAs. In the absence of added salt, *R*_h_ of *L*PMAA was almost ∼1.4-fold larger as compared to eight-arm PMAA of equal molecular weight. An increase in salt concentration to ∼10^–3^ mol/L LiCl resulted in a sharp shrinking of PMAA due to the exponential screening of electrostatic interactions by salt ions.^20,49^ In this salt regime, the magnitude of the salt effect on *R*_h_ was significantly larger for *L*PMAA in comparison to 8PMAA (a 2.1-fold and 1.7-fold decrease, respectively, between low-salt and 0.1 mol/L LiCl). Similarly, the contraction factor *g*_H_, calculated as , was the smallest for eight-arm star polymer and strongly increased with salt concentration (Figure S3). Note that the contraction factor plateaued at LiCl concentrations >0.02 mol/L, and the plateau values of *g*_H_ calculated from the data in Figure S3 (0.85, 0.82, and 0.78 for four-arm, six-arm, and eight-arm PMAA, respectively) were in good agreement with those calculated using the semiempirical equation proposed by Douglas et al. and Shida et al. for neutral polymer stars  (where *f* is the number of arms),^50,51^ which gives the contraction factors of 0.89, 0.79, and 0.72 for *f* = 4, 6, and 8, respectively. The weaker responses of star polyelectrolytes with higher number of arms to changes in salt concentration (Figure 2B and Figure S3) are due to their lower compressibility and higher local salt concentrations caused by redistribution of the salt ions to satisfy the Donnan equilibrium.^9−11^Figure 2B also shows that at salt concentrations *c*_s_ > ∼10^–3^ mol/L LiCl, which largely exceeded the concentration of monomer units of PMAAs *c*_m_ of 1 × 10^–5^ mol/L, the effect of salt on molecular sizes drastically weakened. For this *c*_s_ ≫ *c*_m_ regime, the scaling theory suggests that the radius of gyration of star polyelectrolytes decreases with salt concentration as *R* ∼ *c*_s_^–0.2^. However, fitting the data in Figure 2B for the star polymers above 10^–2^ mol/L LiCl revealed a dependence *R*_h_ ∼ *c*_Li^+^_^–0.11±0.01^ that was weaker than that predicted theoretically (Figure S4). One possible reason for the differences between the theoretical and experimental results is that the theories do not consider counterion condensation. Also note that a lower power law exponent was found in the molecular dynamics simulations (*R* ∼ *c*_s_^–0.16^ vs *R* ∼ *c*_s_^–0.2^ for simulation and theory, respectively).^20^

**Figure 3:**
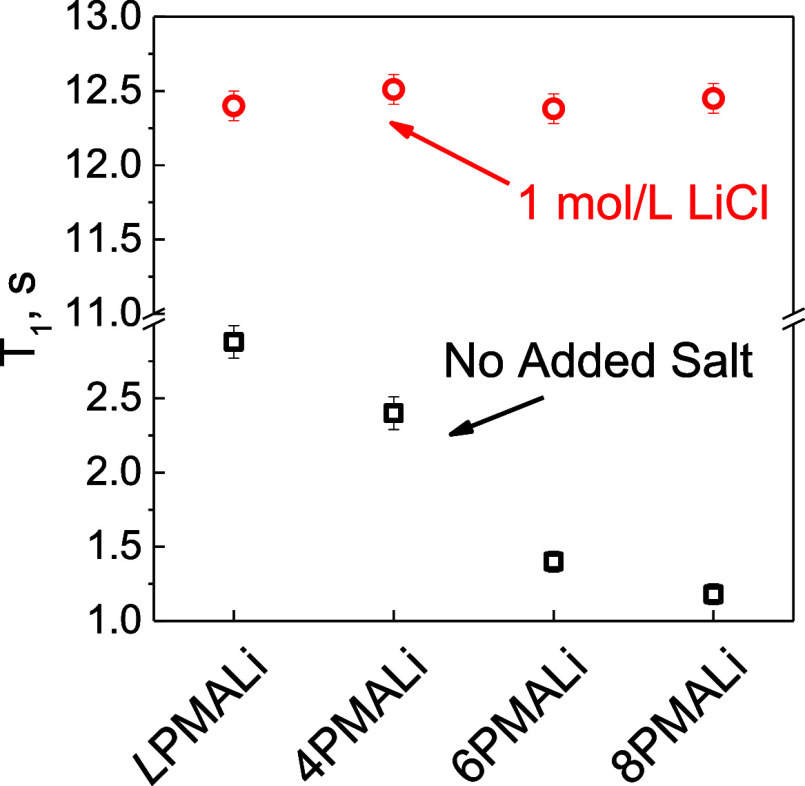
^7^Li spin–lattice relaxation time (*T*_1_) in *c*_m_ = 2.5 × 10^–2^ mol/L *L*PMALi, 4PMALi, 6PMALi, and 8PMALi aqueous solutions containing equimolar concentrations of PMAA units and Li^+^ ions as counterions with no additional salt (squares) and in the presence of 1 mol/L LiCl (circles). All solutions were buffer-free.

We then aimed to probe the distribution of salt counterions between the star or linear polyelectrolytes and the surrounding solution. To that end, we performed ^7^Li NMR measurements with fully ionized PMAA with Li^+^ counterions. These polymethacrylate lithium salts, abbreviated as *L*PMALi, 4PMALi, 6PMALi, and 8PMALi for linear, four-arm star, six-arm star, and eight-arm star, respectively, were prepared by dissolving protonated linear and star PMAAs in a solution of LiOH in which the molar amount of Li^+^ ions was equal to the molar amount of PMAA units. In these solutions, PMAA became ionized, and all Li^+^ ions were present as counterions. Figure S5 shows the ^7^Li NMR spectra of *L*PMALi, 4PMALi, 6PMALi, and 8PMALi solutions that contained no additional salt. While there was no significant changes in the NMR shift between linear and star PMAAs, all peaks were broadened due to ^1^H-^7^Li dipole–dipole interactions.^52^Figure 3 shows ^7^Li NMR spin–lattice relaxation time (*T*_1_) measurements of lithium salts of PMAA to understand the effect of the molecular architecture on the dynamics of Li^+^ ions.

Note that in the absence of added salt, *T*_1_ decreases from 2.8 ± 0.1 s for *L*PMALi to 1.2 ± 0.1 s for 8PMALi. The Bloembergen–Purcell–Pound (BPP) model describes the relationship between spin–lattice relaxation and motional rate. The model is written as
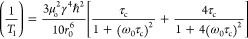
3where γ is the gyromagnetic ratio, *ℏ* is the reduced Planck constant, *r*_o_is the interatomic distance, τ_c_ is the correlation time, and ω_0_ is the Larmor frequency. From this relationship, the ion dynamics of a system can fall in the slow-motion regime (ω_0_τ_c_ ≫ 1) or the fast-motion regimes (ω_0_τ_c_ ≪ 1). For most small polymer molecules, the ion dynamics falls in the fast-motion regime. This can approximate the equation to be
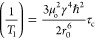
4

A decrease in *T*_1_ gives rise to an increase in τ_c_, reflecting slower ion dynamics.^53,54^ This suggests that in the case of star PMAA, Li^+^ ions are more strongly trapped within the core of the star polymers, while they can move more freely in *L*PMALi solutions. This finding agrees with the computational analysis of Douglas and Chremos that showed reduction in the counterion mobility of star polyelectrolytes compared to their linear counterparts.^55^ Note that the addition of 1 mol/L LiCl increased *T*_1_ to 12.4 s, and at this high salt concentration, the effect of the molecular architecture was no longer observed because the ion dynamics became dominated by the fast-diffusing free Li^+^ ions in solution (Figure 3).

### Self-Diffusion of Linear and Star PMAA in Different Concentration Regimes

**Figure 4:**
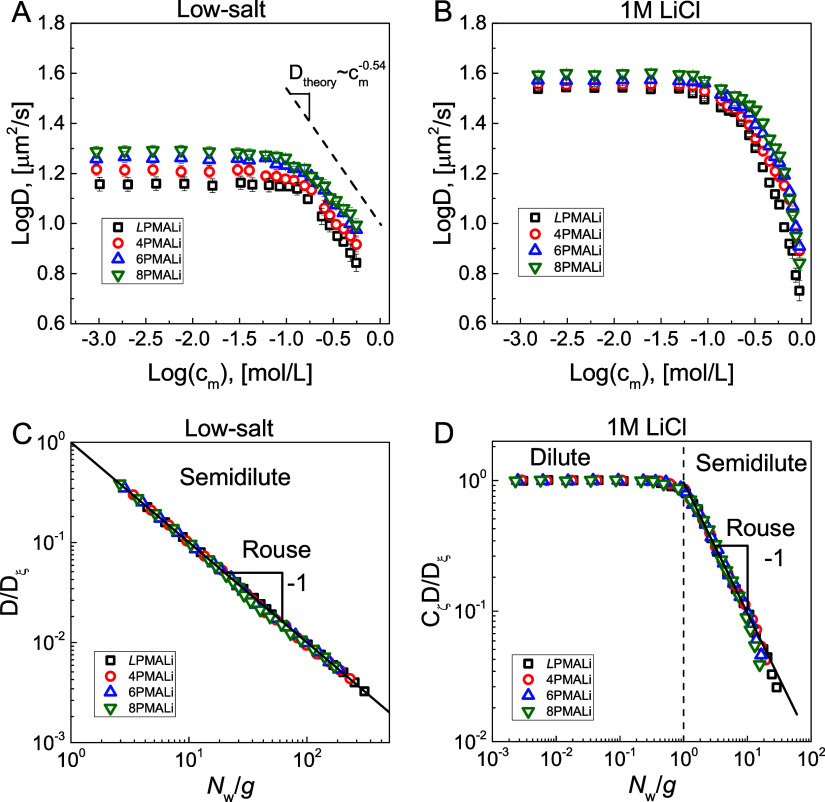
Concentration dependence of the self-diffusion coefficient of polyelectrolyte chains (*L*PMALi (squares)) and stars with different numbers of arms [4PMALi (circles), 6PMALi (triangles), and 8PMALi (inverted triangles)] in 10^–5^ mol/L Tris buffer (A) and 1.0 mol/L LiCl in 10^–5^ mol/L Tris buffer (B) at pH 9. The dashed line shows the theoretical dependence of *D* ∝ *c*_m_^–0.54^ predicted by the polyelectrolyte scaling theory. (C, D) The universal data represent *D*/*D*_ξ_ vs *N*_w_/g for the data sets in (A) and (B), respectively. *D*_ξ_ = *k*_B_*T*/η_s_ξ is a self-diffusion coefficient of the correlation blob, and *g* is the number of repeat units per correlation blob. For the plot in (D), *D*_ξ_/*C*_ζ_ (*C*_ζ_ is a numerical parameter) was set equal to a plateau value in a dilute solution (*c*_m_ < c*) corresponding to . In the calculations of *D*_ξ_, η_s_ of 0.001 Pa · s and 0.00116 Pa · s for low-salt and 1.0 mol/L LiCl concentrations and *T* = 293 K were used.

We then aimed to apply FCS to measure self-diffusion coefficients of linear and star polyelectrolytes over a wide range of polymer concentrations in the two distinct low-salt and high-salt regimes. Although FCS measurements require very low (nanomolar to picomolar) concentrations of fluorescent species, measurements within a wide range of polymer concentrations were enabled by keeping the concentration of labeled PMAA units at the same level and systematically increasing the concentration of unlabeled PMAAs (see Materials and Methods). PMAA labeling with Alexa 488 and FCS measurements was performed as described in our previous publication.^32^Figure 4 compares the concentration dependence of self-diffusion coefficients of linear and star PMALi in 10^–5^ mol/L Tris buffer low-salt solutions (Figure 4A) and 1 mol/L LiCl in 10^–5^ mol/L Tris buffer high-salt solutions (Figure 4B). Tris buffer was added to maintain pH 9 without a significant effect on the distribution of the inorganic counterions. Figure 4 shows that for a fixed polymer concentration, both the low-salt and high-salt data sets exhibit a systematic increase of the diffusion coefficient with increasing number of arms in the star polymers. This trend is expected for PMAAs of equal molecular weight as the compactness of polymers increases with polymer branching. Moreover, both data sets have plateaus at low polymer concentrations and show slowing of the polymer diffusion at higher polymer concentrations. However, as will be evident from the discussion below, the plateaus in Figure 4A,B are fundamentally different in nature.

To understand the overall concentration dependences of polyelectrolyte diffusion, we employed the scaling theory of polyelectrolyte solutions.^49,56,57^ This approach suggests that in semidilute polyelectrolyte solutions, chains are represented by the correlation blobs of length ξ (known as the solution correlation length). Each blob consists of *g* repeat units, which are related to concentration of polymer units *c*_m_ and ξ as *g* ≈ *c*_m_ξ^3^. Within the correlation blob, the chain statistics is governed by the polymer–polymer and polymer–solvent interactions, while on the length scales larger than ξ interactions are screened, and a polymer chain behaves as an ideal chain made of correlation blobs.^49,56^ The concentration dependence of the solution correlation length is determined by the type of dominant interactions on the length scales *r* ∝ ξ as illustrated in Figure S6.^49,56,58^Figure S6 introduces an overlap concentration of electrostatic blobs, *c*_D_, representing the length scale at which the energy of electrostatic interactions between ionized groups on the polymer backbone is comparable to thermal energy. In the case of low polyelectrolyte and salt concentrations (applicable to the plateau region in Figure 4A, at *c*_m_ < *c*_D_), the electrostatic repulsion between the ionized groups on the polymer backbone results in chain stretching so that . At polymer concentrations *c*_m_ > *c*_D_, transition to a new regime occurs, in which the electrostatic interactions are screened and solution properties of polyelectrolytes become similar to those of neutral polymers. In this regime, ξ ∝ *c*_m_^ν/(1–3ν)^, with the exponent ν being determined by the solvent quality for the polymer chains. Finally, at high salt concentrations, polyelectrolytes behave as neutral polymers with the effective two-body interactions whose strength is controlled by salt concentration.^49,56^

The scaling theory also predicts that self-diffusion coefficient *D* of polyelectrolyte chains with a repeat unit projection length *l* and number of units *N* in a solvent with viscosity η_s_ is related to concentration of polymer units *c*_m_ through the following equation:^49,56^
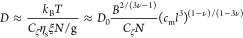
5where *k*_B_ is the Boltzmann constant, *T* is the absolute temperature, *C*_ζ_ is a numerical coefficient equal to 6π and ≈5 for hard spheres and for the Rouse model of an ideal chain, respectively, and *D*_0_ is a repeat unit diffusion coefficient equal to , where η_s_ is the solvent viscosity and *l* is the repeat unit projection length in the all-trans chain conformation equal to 0.255 nm.^59^ Determination of *B*-parameters in eq 5 for low-salt and high-salt regimes is detailed in the Supporting Information and shown in Tables S1 and S2 for the two salt regimes, respectively. In this work, we applied the scaling theory to polyelectrolyte stars, assuming that a similar concentration dependence of self-diffusion coefficient is expected in semidilute solutions of overlapping polyelectrolyte stars with a small number of arms.

At low salt concentration, the plateau in Figure 4A corresponds to a polyelectrolyte regime occurring at *c*_m_ < *c*_D_ (Figure S6). In this regime, strong electrostatic interactions result in ξ ∝ g ∝ *c*_m_^–1/2^ and ν = 1. Substituting ν = 1 into eq 5 leads to *D* ∝ *c*^0^, as observed in the plateau region in Figure 4A. A departure from the plateau indicates a crossover to semidilute solutions of the overlapping electrostatic blobs with swollen star arms, leading to the predicted ξ ∝ g^0.588^ ∝ *c*_m_^–0.77^ and *D* ∝ *c*_m_^–0.54^ dependences.

We then aimed to uncover the underlying universal scaling behavior of linear and star PMAAs by constructing a master curve. To that end, the FCS data on self-diffusion in Figure 4A were normalized using the diffusion coefficient of the correlation blob *D*_ξ_ = *k*_B_*T*/η_s_ξ as a normalization factor. Using the calculated *B*_pe_ and *B*_g_ parameters (as detailed in the Supporting Information and shown in Table S1), the FCS data were replotted as *D*/*D*_ξ_ vs *N*_w_/g (Figure 4C), where *N*_w_ is number of repeat units per chain. The additional plots of the normalized diffusion coefficients of PMALi polyelectrolytes in the low-salt regime are shown in Figure S7. Also note that the polyelectrolyte scaling analysis also enabled calculation of the overlap concentration of electrostatic blobs *c*_D_ for linear and star PMAA, yielding higher *c*_D_ for stars compared to linear PMAA (Table S1). Importantly, at low-salt concentrations, all the data sets for the polyelectrolyte chains and stars with different numbers of arms collapsed into a single universal curve, suggesting that an unentangled Rouse regime covered the entire polymer concentration range.

While the experiments shown Figure 4 and Figure S7 employed PMAAs* with Li^+^ counterions, a similar behavior was observed for linear and star polymethacrylate Na^+^ and K^+^ salts (PMANa and PMAK) at the low-salt regime (Figures S8 and S9). Specifically, while the values of self-diffusion coefficients of PMAA slightly varied for different types of counterions, reflecting the Hofmeister effect, as in the case of the polyelectrolytes with Li^+^ counterions, all of the data also collapsed into a single universal curve for Na^+^ and K^+^ counterions. This suggests that the mechanism of polymer diffusion for linear and star polyelectrolytes is similar and independent of the chemistry of counterions.

Scaling analysis also allowed us to estimate the number of polyelectrolyte units per correlation blob *g*. The data shown in Figure S10 suggests that in low-salt solutions, *g* decreased at high polymer concentrations, with a stronger decrease at concentrations *c*_m_ > *c*_D_. For all counterions, *g* systematically increased with polymer branching, reaching twofold larger values for eight-arm star polyelectrolytes as compared to their linear counterpart. The larger correlation blobs of star polyelectrolytes agrees with the suggestion of a larger persistence length of star polyelectrolytes due to higher segmental density and the constrains introduced by the branching point in star polymers.^23^

At high salt concentration *c*_s_ = 1.0 mol/L (Figure 4B), the plateau in the self-diffusion coefficient corresponds to a dilute solution regime. Exponential screening of the electrostatic interactions reduced their effect to effective short-range interactions, leading to renormalization of solvent quality for the polymer backbone. A decrease in the self-diffusion coefficient at high polymer concentrations reflects a crossover to the semidilute solution regime. In this concentration range, we can identify good and theta solvent behavior of the solution correlation length with characteristic exponents for concentration dependence of the self-diffusion coefficient *D* ∝ *c*_m_^–0.54^ and *D* ∝ *c*_m_^–1^ dependences, respectively. Extracting *B*-parameters from the experimental data using eq 5 and replotting the diffusion data in the universal form (see Table S2 for details) allowed us to overlap the data sets for linear and star polyelectrolytes within the entire concentration range spanning both dilute and semidilute solutions. For this plot, we expanded concentration dependence of *g* into a dilute solution regime (*N*/*g* ≪ 1) and set *D*_ξ_/*C*_ζ_ equal to the plateau value (Figure 4D). At high polymer concentrations (*N*/g ≫ 1), we see a deviation of the data sets from the expected Rouse scaling of the diffusion coefficient, likely indicating a crossover to solution of entangled linear chains and stars (Figure S11).

## Conclusion and Outlook

This work explored dynamics of polyelectrolytes of different molecular topology (linear vs star) in solutions at different pH, salt, and polymer concentrations. In dilute solutions, measurements of hydrodynamic size reflected a more compact structure of star polyelectrolytes as compared to linear counterparts and a lower magnitude of response of molecular size to changes in pH and/or concentration of salt. The use of different monovalent cations (Li^+^, Na^+^, K^+^, and Cs^+^) in salt cations of monovalent salts confirmed the significant role of a counterion in molecular diffusion and was consistent with the Hofmeister effect on ionic binding. Importantly, this work reports direct experimental evidence for stronger binding of counterions within star polymers compared to linear polyelectrolyte chains. This conclusion was made by performing ^7^Li NMR experiments, which indicated lower mobility of Li^+^ counterions within star polyelectrolytes due to their higher local charge density.

By applying the scaling theory to analysis of the FCS data on diffusion of semidilute solutions of linear and star polyelectrolytes with different numbers of arms in a wide range of polymer concentration, this work identified different origins of polymer concentration regimes at low and high salt conditions. In low-salt solutions, a broad concentration range occurred with the self-diffusion coefficient being concentration independent and increasing with the number of arms. At low polymer concentrations, the scaling relationship *D* ∝ *c*_m_^0^ was observed, while at higher polymer concentrations, *D* decreased with polymer concentration as expected in semidilute solutions of the overlapping electrostatic blobs. Both findings indicate that linear and star polyelectrolytes show unentangled Rouse-like dynamics in a wide range of polyelectrolyte concentration (10^–3^ mol/L< *c*_m_ < 0.5 mol/L). In contrast, at high salt concentrations, electrostatic interactions are screened and polyelectrolyte chains and stars behave as their neutral counterparts. In agreement with the current understanding of entanglements in polyelectrolyte solutions, a crossover to entangled solution regime was observed only in high-salt solutions of polyelectrolytes.^58^

Note that while the scaling theory for linear polyelectrolytes can adequately be used for the description of the behavior of star polymers, there were also some discrepancies between theory and the experiment. For example, the scaling theory suggests that *D* ∝ *c*_m_^–0.54^ at *c*_m_ > *c*_D_ (Figure S6), while the experimentally observed slopes were smaller and decreased from −0.48 for PMALi to −0.37 for 8PMALi (Figure S12, *R*^2^ 0.989–0.995). This deviation was probably due to the effect of polymer architecture on interactions of polyelectrolytes with a solvent and the effect of excluded volume interactions on diffusion of star polymers.^60^ This suggests that further experiments and theory development are required for a better understanding of the dynamics of branched polyelectrolytes in solutions.

## Supporting Information Available

Data on polymer diffusion in solutions at different polymer and salt concentrations, detailed scaling theory of polyelectrolytes and its application to the experimental data (PDF)

## References

[ref1] Ren J. M.; McKenzie T. G.; Fu Q.; Wong E. H. H.; Xu J.; An Z.; Shanmugam S.; Davis T. P.; Boyer C.; Qiao G. G. Star Polymers. Chem. Rev. 2016, 116 (12), 6743–6836. 10.1021/acs.chemrev.6b00008. 27299693

[ref2] Chremos A.; Douglas J. F. Solution properties of star polyelectrolytes having a moderate number of arms. J. Chem. Phys. 2017, 147 (4), 044906 10.1063/1.4995534. 28764357 PMC5702915

[ref3] Fan J.; Emamy H.; Chremos A.; Douglas J. F.; Starr F. W. Dynamic heterogeneity and collective motion in star polymer melts. J. Chem. Phys. 2020, 152 (5), 054904 10.1063/1.5135731. 32035474

[ref4] Chremos A.; Glynos E.; Green P. F. Structure and dynamical intra-molecular heterogeneity of star polymer melts above glass transition temperature. J. Chem. Phys. 2015, 142 (4), 044901 10.1063/1.4906085. 25638003

[ref5] Lue L.; Kiselev S. B. Star Polymers in Good Solvents from Dilute to Concentrated Regimes: Crossover Approach. Condensed Matter Physics 2002, 5, 73–104. 10.5488/CMP.5.1.73.

[ref6] Robello D. R.; André A.; McCovick T. A.; Kraus A.; Mourey T. H. Synthesis and Characterization of Star Polymers Made from Simple. Multifunct. Initiators. Macromol. 2002, 35 (25), 9334–9344. 10.1021/ma0206570.

[ref7] van Ruymbeke E.; Coppola S.; Balacca L.; Righi S.; Vlassopoulos D. Decoding the viscoelastic response of polydisperse star/linear polymer blends. J. Rheol. 2010, 54 (3), 507–538. 10.1122/1.3368729.

[ref8] Borisov O. V. Conformations of Star-Branched Polyelectrolytes. J. Phys. II France 1996, 6 (1), 1–19. 10.1051/jp2:1996164.

[ref9] Borisov O. V.; Zhulina E. B. Effects of ionic strength and charge annealing in star-branched polyelectrolytes. European Physical Journal B - Condensed Matter and Complex Systems 1998, 4 (2), 205–217. 10.1007/s100510050371.

[ref10] Klein Wolterink J.; Leermakers F. A. M.; Fleer G. J.; Koopal L. K.; Zhulina E. B.; Borisov O. V. Screening in Solutions of Star-Branched Polyelectrolytes. Macromolecules 1999, 32 (7), 2365–2377. 10.1021/ma981501w.

[ref11] Klein Wolterink J.; van Male J.; Cohen Stuart M. A.; Koopal L. K.; Zhulina E. B.; Borisov O. V. Annealed Star-Branched Polyelectrolytes in Solution. Macromolecules 2002, 35 (24), 9176–9190. 10.1021/ma020781j.

[ref12] Klein Wolterink J.; van Male J.; Daoud M.; Borisov O. V. Starburst Polyelectrolytes: Scaling and Self-Consistent-Field Theory. Macromolecules 2003, 36 (17), 6624–6631. 10.1021/ma030187p.

[ref13] Leermakers F. A. M.; Ballauff M.; Borisov O. V. Counterion Localization in Solutions of Starlike Polyelectrolytes and Colloidal Polyelectrolyte Brushes: A Self-Consistent Field Theory. Langmuir 2008, 24 (18), 10026–10034. 10.1021/la8013249. 18698859

[ref14] Polotsky A. A.; Zhulina E. B.; Birshtein T. M.; Borisov O. V. Effect of the Ionic Strength on Collapse Transition in Star-like Polyelectrolytes. Macromol. Symp. 2009, 278 (1), 24–31. 10.1002/masy.200950404.

[ref15] Košovan P.; Kuldová J.; Limpouchová Z.; Procházka K.; Zhulina E. B.; Borisov O. V. Molecular dynamics simulations of a polyelectrolyte star in poor solvent. Soft Matter 2010, 6 (9), 1872–1874. 10.1039/b925067k.

[ref16] Polotsky A. A.; Zhulina E. B.; Birshtein T. M.; Borisov O. V. Collapse of a weak polyelectrolyte star in a poor solvent. Soft Matter 2012, 8 (36), 9446–9459. 10.1039/c2sm25593f.

[ref17] Uhlík F.; Košovan P.; Limpouchová Z.; Procházka K.; Borisov O. V.; Leermakers F. A. M. Modeling of Ionization and Conformations of Starlike Weak Polyelectrolytes. Macromolecules 2014, 47 (12), 4004–4016. 10.1021/ma500377y.

[ref18] Uhlík F.; Košovan P.; Zhulina E. B.; Borisov O. V. Charge-controlled nano-structuring in partially collapsed star-shaped macromolecules. Soft Matter 2016, 12 (21), 4846–4852. 10.1039/C6SM00109B. 27140226

[ref19] Shusharina N. P.; Rubinstein M. Concentration Regimes in Solutions of Polyelectrolyte Stars. Macromolecules 2008, 41 (1), 203–217. 10.1021/ma0711442.

[ref20] Jusufi A.; Likos C. N. Colloquium: Star-branched polyelectrolytes: The physics of their conformations and interactions. Rev. Mod. Phys. 2009, 81 (4), 1753–1772. 10.1103/RevModPhys.81.1753.

[ref21] Furukawa T.; Ishizu K. Synthesis and Viscoelastic Behavior of Multiarm Star Polyelectrolytes. Macromolecules 2005, 38 (7), 2911–2917. 10.1021/ma047777n.

[ref22] Moinard D.; Taton D.; Gnanou Y.; Rochas C.; Borsali R. SAXS from Four-Arm Polyelectrolyte Stars in Semi-Dilute Solutions. Macromol. Chem. Phys. 2003, 204 (1), 89–97. 10.1002/macp.200290061.

[ref23] Moinard D.; Borsali R.; Taton D.; Gnanou Y. Scattering and Viscosimetric Behaviors of Four- and Six-Arm Star Polyelectrolyte Solutions. Macromolecules 2005, 38 (16), 7105–7120. 10.1021/ma050505f.

[ref24] Heinrich M.; Rawiso M.; Zilliox J. G.; Lesieur P.; Simon J. P. Small-angle X-ray scattering from salt-free solutions of star-branched polyelectrolytes. Eur. Phys. J. E 2001, 4 (2), 131–142. 10.1007/s101890170122.

[ref25] Shew C. Y.; Do C.; Hong K.; Liu Y.; Porcar L.; Smith G. S.; Chen W. R. Conformational effect on small angle neutron scattering behavior of interacting polyelectrolyte solutions: A perspective of integral equation theory. J. Chem. Phys. 2012, 137 (2), 024907 10.1063/1.4732516. 22803562

[ref26] Boué F.; Combet J.; Demé B.; Heinrich M.; Zilliox J.-G.; Rawiso M. SANS from Salt-Free Aqueous Solutions of Hydrophilic and Highly Charged Star-Branched Polyelectrolytes. Polymers 2016, 8 (6), 228 10.3390/polym8060228. 30979321 PMC6431935

[ref27] Furukawa T.; Uchida S.; Ishizu K. Synthesis and polyelectrolyte behavior of poly(methacrylic acid) star polymers. J. Appl. Polym. Sci. 2007, 105 (3), 1543–1550. 10.1002/app.24966.

[ref28] Plamper F. A.; Becker H.; Lanzendörfer M.; Patel M.; Wittemann A.; Ballauff M.; Müller A. H. E. Synthesis, Characterization and Behavior in Aqueous Solution of Star-Shaped Poly(acrylic acid). Macromol. Chem. Phys. 2005, 206 (18), 1813–1825. 10.1002/macp.200500238.

[ref29] Witten T. A.; Pincus P. A.; Cates M. E. Macrocrystal Ordering in Star Polymer Solutions. Europhys. Lett. 1986, 2 (2), 137 10.1209/0295-5075/2/2/011.

[ref30] Ishizu K.; Ono T.; Uchida S. Structural Ordering in Star Polymer Solutions. Polym.-Plast. Technol. Eng. 1997, 36 (3), 461–471. 10.1080/03602559708000635.

[ref31] Pristinski D.; Kozlovskaya V.; Sukhishvili S. A. Fluorescence correlation spectroscopy studies of diffusion of a weak polyelectrolyte in aqueous solutions. J. Chem. Phys. 2004, 122 (1), 014907 10.1063/1.1829255. 15638700

[ref32] Aliakseyeu A.; Shah P. P.; Ankner J. F.; Sukhishvili S. A. Salt-Induced Diffusion of Star and Linear Polyelectrolytes within Multilayer Films. Macromolecules 2023, 56 (14), 5434–5445. 10.1021/acs.macromol.3c00777. 38357536 PMC10863069

[ref33] Sukhishvili S. A.; Chen Y.; Müller J. D.; Gratton E.; Schweizer K. S.; Granick S. Surface Diffusion of Poly(ethylene glycol). Macromolecules 2002, 35 (5), 1776–1784. 10.1021/ma0113529.

[ref34] Yu L.; Lei Y.; Ma Y.; Liu M.; Zheng J.; Dan D.; Gao P. A Comprehensive Review of Fluorescence Correlation Spectroscopy. Front. Phys. 2021, 9, 644450 10.3389/fphy.2021.644450.

[ref35] Rumble J. CRC Handbook of Chemistry and Physics. CRC Press: 2023.

[ref36] LeBel R. G.; Goring D. A. I. Density, Viscosity, Refractive Index, and Hygroscopicity of Mixtures of Water and Dimethyl Sulfoxide. Journal of Chemical & Engineering Data 1962, 7 (1), 100–101. 10.1021/je60012a032.

[ref37] Kharlampieva E.; Pristinski D.; Sukhishvili S. A. Hydrogen-Bonded Multilayers of Poly(carboxybetaine)s. Macromolecules 2007, 40 (19), 6967–6972. 10.1021/ma071152i.

[ref38] Aliakseyeu A.; Ankner J. F.; Sukhishvili S. A. Impact of Star Polyacid Branching on Polymer Diffusion within Multilayer Films. Macromolecules 2022, 55 (18), 8150–8161. 10.1021/acs.macromol.2c01104.

[ref39] Ohno H.; Abe K.; Tsuchida E. Solvent effect on the formation of poly(methacrylic acid)-poly(N-vinyl-2-pyrrolidone) complex through hydrogen bonding. Makromol. Chem. 1978, 179 (3), 755–763. 10.1002/macp.1978.021790318.

[ref40] Selin V.; Aliakseyeu A.; Ankner J. F.; Sukhishvili S. A. Effect of a Competitive Solvent on Binding Enthalpy and Chain Intermixing in Hydrogen-Bonded Layer-by-Layer Films. Macromolecules 2019, 52 (12), 4432–4440. 10.1021/acs.macromol.9b00650.

[ref41] Hofmeister F. Zur Lehre von der Wirkung der Salze. Archiv für experimentelle Pathologie und Pharmakologie 1888, 24 (4), 247–260. 10.1007/BF01918191.

[ref42] Gregory K. P.; Wanless E. J.; Webber G. B.; Craig V. S. J.; Page A. J. The electrostatic origins of specific ion effects: quantifying the Hofmeister series for anions. Chemical Science 2021, 12 (45), 15007–15015. 10.1039/D1SC03568A. 34976339 PMC8612401

[ref43] Gregory K. P.; Elliott G. R.; Robertson H.; Kumar A.; Wanless E. J.; Webber G. B.; Craig V. S. J.; Andersson G. G.; Page A. J. Understanding specific ion effects and the Hofmeister series. Phys. Chem. Chem. Phys. 2022, 24 (21), 12682–12718. 10.1039/D2CP00847E. 35543205

[ref44] Moghaddam S. Z.; Thormann E. The Hofmeister series: Specific ion effects in aqueous polymer solutions. J. Colloid Interface Sci. 2019, 555, 615–635. 10.1016/j.jcis.2019.07.067. 31408761

[ref45] Kang B.; Tang H.; Zhao Z.; Song S. Hofmeister Series: Insights of Ion Specificity from Amphiphilic Assembly and Interface Property. ACS Omega 2020, 5 (12), 6229–6239. 10.1021/acsomega.0c00237. 32258857 PMC7114165

[ref46] Masiak M.; Hyk W.; Stojek Z.; Ciszkowska M. Structural Changes of Polyacids Initiated by Their Neutralization with Various Alkali Metal Hydroxides. Diffusion Studies in Poly(acrylic acid)s. J. Phys. Chem. B 2007, 111 (38), 11194–11200. 10.1021/jp0711904. 17760434

[ref47] Požar J.; Bohinc K.; Vlachy V.; Kovačević D. Ion-specific and charge effects in counterion binding to poly(styrenesulfonate) anions. Phys. Chem. Chem. Phys. 2011, 13 (34), 15610–15618. 10.1039/c1cp21291e. 21792404

[ref48] May C. E.; Philipp W. H. Ion exchange selectivity for cross-linked polyacrylic acid; NTRS - NASA Technical Reports Server 1983.

[ref49] Dobrynin A. V.; Colby R. H.; Rubinstein M. Scaling Theory of Polyelectrolyte Solutions. Macromolecules 1995, 28 (6), 1859–1871. 10.1021/ma00110a021.

[ref50] Douglas J. F.; Roovers J.; Freed K. F. Characterization of branching architecture through ″universal″ ratios of polymer solution properties. Macromolecules 1990, 23 (18), 4168–4180. 10.1021/ma00220a022.

[ref51] Shida K.; Ohno K.; Kawazoe Y.; Nakamura Y. Hydrodynamic factors for linear and star polymers on lattice under the theta condition. Polymer 2004, 45 (5), 1729–1733. 10.1016/j.polymer.2003.12.063.

[ref52] Stallworth P. E.; Greenbaum S. G.; Croce F.; Slane S.; Salomon M. Lithium-7 NMR and ionic conductivity studies of gel electrolytes based on poly(methylmethacrylate). Electrochim. Acta 1995, 40 (13), 2137–2141. 10.1016/0013-4686(95)00153-6.

[ref53] Karimineghlani P.; Zheng J.; Hu Y.-Y.; Sukhishvili S. Solvation and diffusion of poly(vinyl alcohol) chains in a hydrated inorganic ionic liquid. Phys. Chem. Chem. Phys. 2020, 22 (31), 17705–17712. 10.1039/D0CP02679D. 32728682

[ref54] Adebahr J.; Forsyth M.; MacFarlane D. R.; Gavelin P.; Jacobsson P. Lithium coordination and mobility in gel electrolytes based on an acrylate polymer with ethylene oxide side chains. J. Mater. Chem. 2003, 13 (4), 814–817. 10.1039/b208354j.

[ref55] Chremos A.; Douglas J. F. Counter-ion distribution around flexible polyelectrolytes having different molecular architecture. Soft Matter 2016, 12 (11), 2932–2941. 10.1039/C5SM02873F. 26864861

[ref56] Dobrynin A. V.; Rubinstein M. Theory of polyelectrolytes in solutions and at surfaces. Prog. Polym. Sci. 2005, 30 (11), 1049–1118. 10.1016/j.progpolymsci.2005.07.006.

[ref57] De Gennes P. G. Dynamics of Entangled Polymer Solutions. I. The Rouse Model. Macromolecules 1976, 9 (4), 587–593. 10.1021/ma60052a011.

[ref58] Dobrynin A. V.; Jacobs M. When Do Polyelectrolytes Entangle?. Macromolecules 2021, 54 (4), 1859–1869. 10.1021/acs.macromol.0c02450.

[ref59] Doi M.; Edwards S. F. The Theory of Polymer Dynamics. Clarendon Press: 1986.

[ref60] Singh S. P.; Huang C.-C.; Westphal E.; Gompper G.; Winkler R. G. Hydrodynamic correlations and diffusion coefficient of star polymers in solution. J. Chem. Phys. 2014, 141 (8), 084901 10.1063/1.4893766. 25173039

