# Supplementary material for: Self-Diffusion of Star and Linear Polyelectrolytes in Salt-Free and Salt Solutions
Source: Macromolecules. 2024 Dec 27;58(1):240–8. doi: 10.1021/acs.macromol.4c01374 (PMC11741142; doi:10.1021/acs.macromol.4c01374)
Supplement: Supplementary file 1 — ma4c01374_si_001.pdf [file ma4c01374_si_001.pdf]

SUPPORTING INFORMATION

Self-diffusion of Star and Linear Polyelectrolytes in Salt-Free and Salt Solutions

Aliaksei Aliakseyeu,[1,2] Erica Truong[3], Yan-Yan Hu,[3,4] Ryan Sayko,[5] Andrey Dobrynin[5]* and Svetlana A. Sukhishvili[1]*

[1]Department of Materials Science & Engineering, Texas A&M University, College Station, TX 77840, USA

[2]Department of Chemical Engineering, Texas A&M University, College Station, TX 77840, USA

*svetlana@tamu.edu

[3]Department of Chemistry and Biochemistry, Florida State University, Tallahassee, FL 32306, USA

[4]Center of Interdisciplinary Magnetic Resonance, the National High Magnetic Field Laboratory, 1800 East Paul Dirac Drive, Tallahassee, FL 32310, USA

[5]Department of Chemistry, University of North Carolina, Chapel Hill, NC 27599-3290, USA

*avd@email.unc.edu

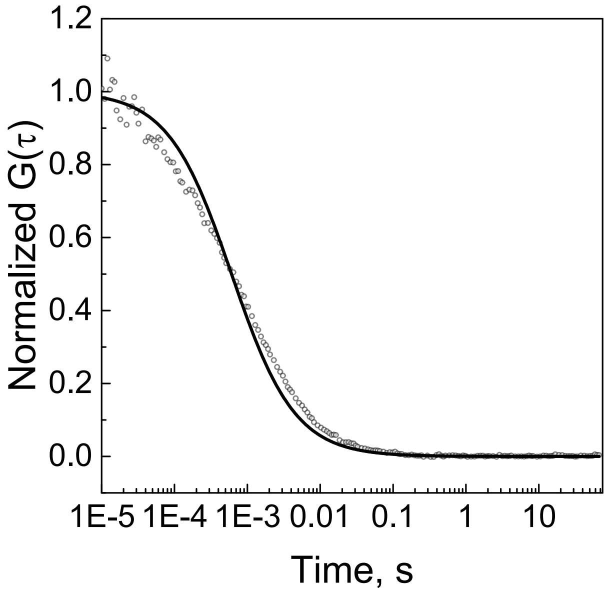

Fig. S1. The normalized autocorrelation function measured for diffusion of the equimolar mixture of 10^{-3} mg/ml *L*PMAA* and 10^{-8} mol/L Alexa 488 in 0.01 mol/L sodium phosphate buffer at pH 9 and 20 ^{o}C. The data were fitted with the single component 3D diffusion equation (1) shown in the main text.

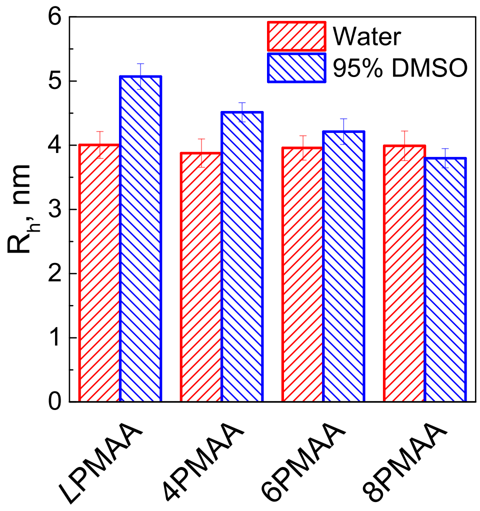

Fig. S2. The hydrodynamic radii of linear and star PMAAs measured in 10^{-5} mol/L aqueous solutions at pH 3 (red) and in a 95 vol% DMSO/5 vol% water solvent (blue, viscosity of the mixture $\eta_s = 0.00229$ Pa $\cdot$ s).[3] Temperature was 20 ^{o}C.

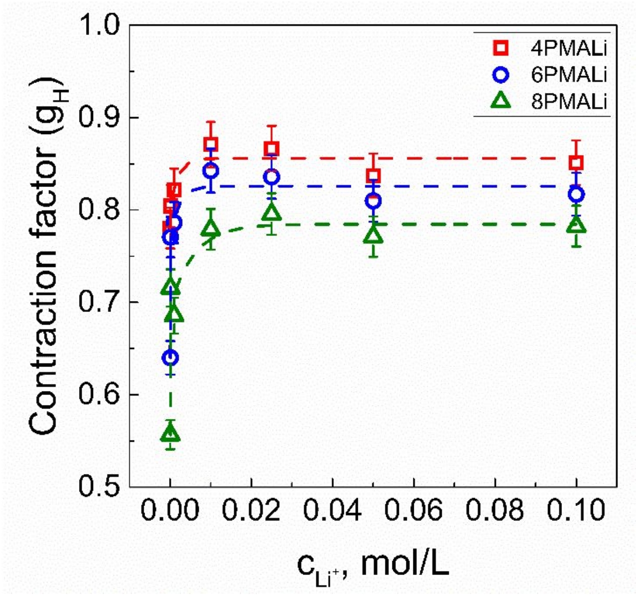

Fig. S3. The hydrodynamic contraction factor g_H, calculated as $\left(R_h^{star}/R_h^{linear}\right)^2$, for 4PMA*Li (red squares), 6PMA*Li (blue circles) and 8PMA*Li (olive triangles) in 10^{-5} mol/L solutions as a function of LiCl concentration.

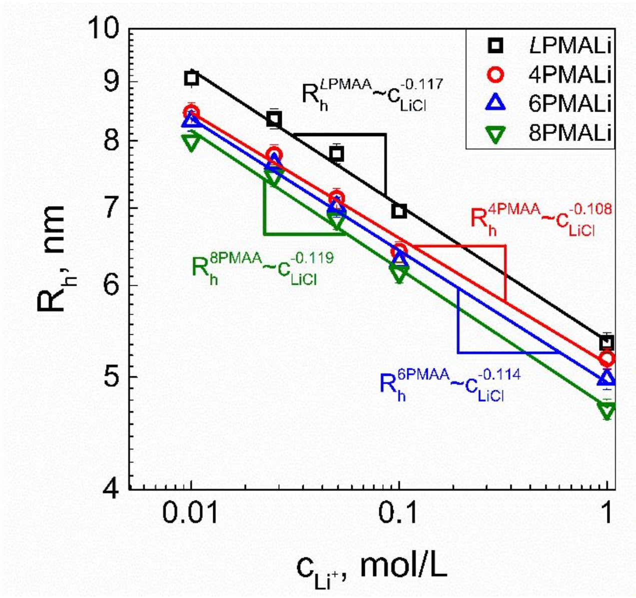

Fig. S4. The hydrodynamic radii of *L*PMALi (squares), 4PMALi (circles), 6PMALi (triangles) and 8PMALi (inverted triangles) as a function of Li^+ concentration. The lines represent the power law fitting to the following equation: $R_h \sim c_s^a$. All solutions contained 10^{-5} mol/L PMAA in $1*10^{-5}$ mol/L Tris buffer at pH=9. The hydrodynamic radii were calculated as $R_h = k_B T/6\pi\eta_s D$ using the solution viscosity η_s values calculated for different concentration of LiCl.[4]

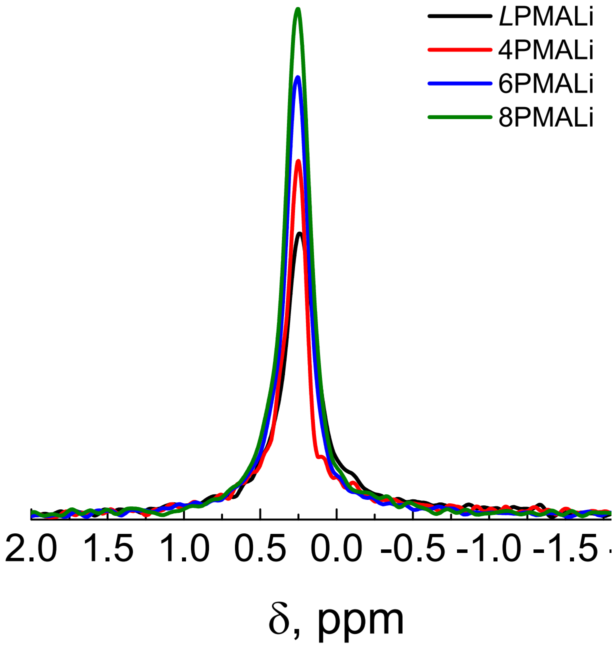

Fig. S5. ^{7}Li NMR spectra of linear and star PMALi at the equimolar concentrations of the lithium ions and the carboxylate groups of PMAAs of 25 mmol/L each.

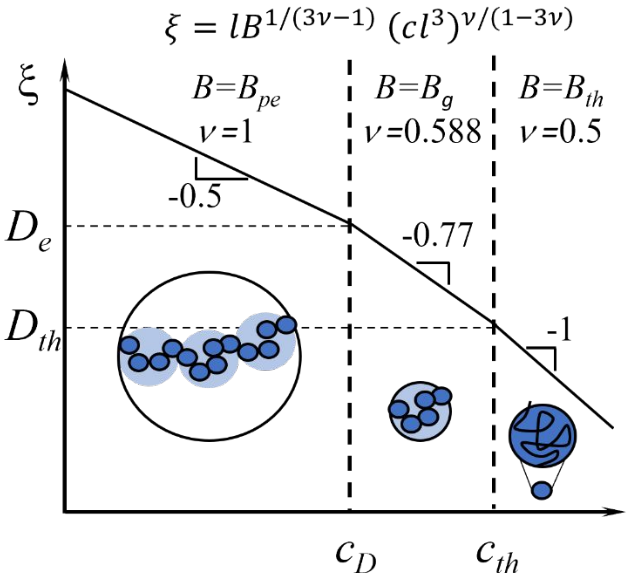

Fig. S6. The log-log plot of the correlation length vs polymer concentration in semidilute polyelectrolytes solutions. Different solution regimes are separated by the crossover concentration c_D (overlap concentration of electrostatic blobs) and c_{th} (overlap concentration of thermal blobs). The B-parameters characterize chain properties on different length scales and l is the repeat unit projection length in the all-trans conformation.[5]

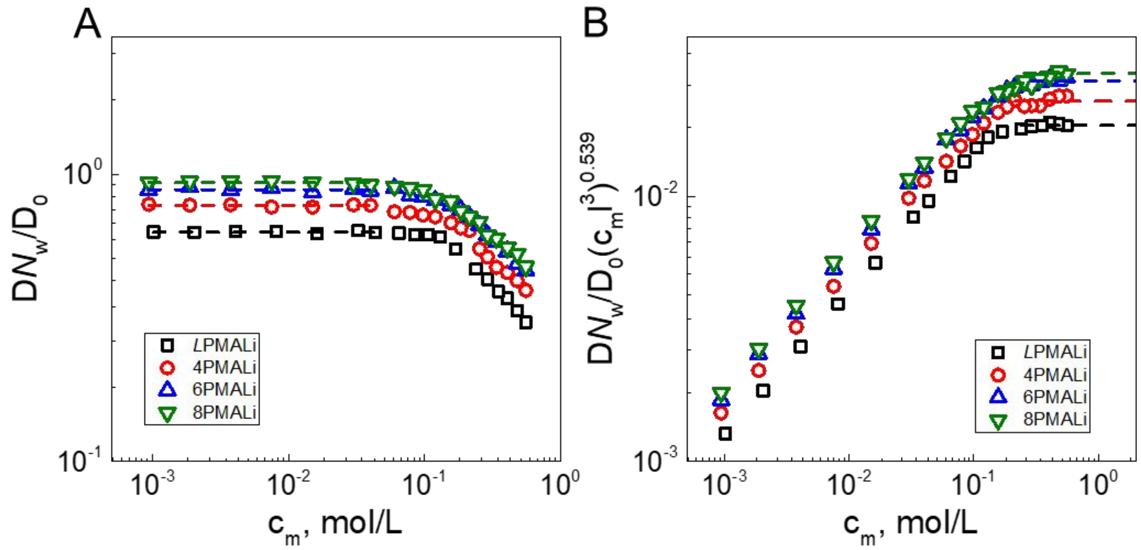

Fig. S7. The log-log dependences of normalized diffusion coefficient DN_w/D_0 (A) and DN_w/D_0 $(c_m l^3)^{0.539}$ (B) on polymer concentration in low salt solutions of LiCl for linear PMALi (black squares), 4PMALi (red circles), 6PMALi (blue triangles), and 8PMALi (green inverted triangles) in $1*10^{-5}$ mol/L Tris buffer at pH=9. Dashed lines in (A) correspond to estimated values of B_{pe}/C_ζ equal to 0.629 (black), 0.779 (red), 0.886 (blue), and 0.936 (green). Dashed lines in (B) correspond to the estimated values of $C_{p,\mathrm{g}}$ equal to 0.019 (black), 0.024 (red), 0.028 (blue), and 0.029 (green). $D_0 = k_B T/\eta_s l = 1.59 \times 10^{-8} m^2/s$, $T =$ 293.15 K, solvent viscosity $\eta_s = 0.001 \mathrm{Pa} \cdot \mathrm{s}$ for low-salt concentration and $l = 0.255$ nm is the monomer projection length in the all-trans conformation.

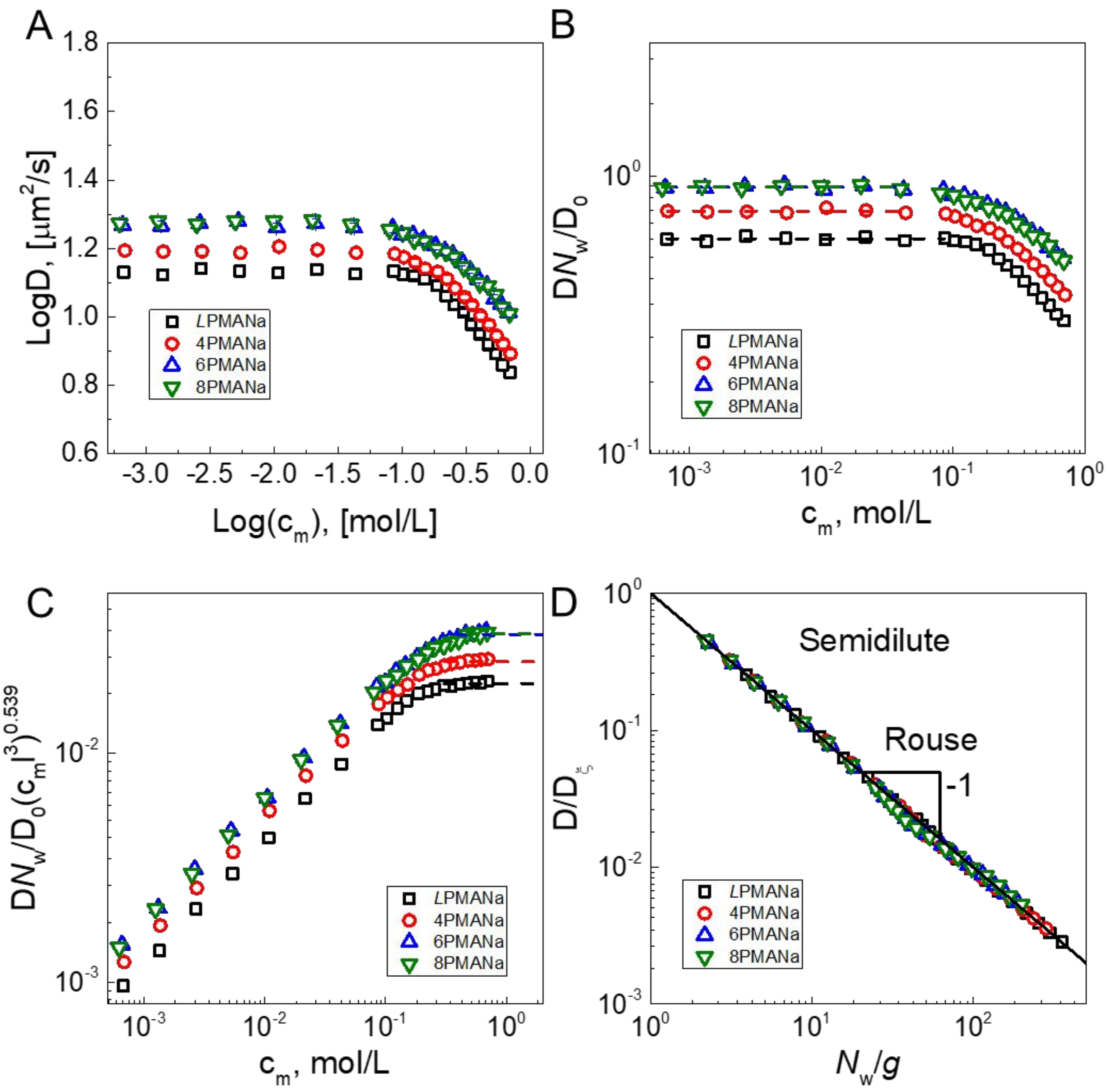

Fig. S8. The dependences of diffusion coefficient D (A), as well as the normalized diffusion coefficients $D N_w/D_0$ (B) and $DN_w/D_0(c_m l^3)^{0.539}$(C) on polymer unit concentration in low-salt solutions for linear *L*PMANa (black squares), 4PMANa (red circles), 6PMANa (blue triangles), and 8PMANa (green inverted triangles) in $1*10^{-5}$ mol/L Tris buffer at pH=9. The dashed lines in (B) show the estimated values of B_{pe}/C_ζ equal to 0.594 (black), 0.744 (red), 0.906 (blue), and 0.912 (green). The dashed lines in (C) correspond to the estimated values of $C_{p,\mathrm{g}}$ equal to 0.020 (black), 0.026 (red), 0.033 (blue), and 0.033 (green). The normalized diffusion coefficient D/D_ξ as a function of number of correlation blobs N_w/g (D), with the solid line indicating a scaling dependence in the Rouse regime. $D_0 = k_B T/\eta_s l$= $1.59 \times 10^{-8} m^2/s$, $T =$ 293.15 K, solvent viscosity $\eta_s = 0.001$ Pa · s, and $l = 0.255$ nm is the monomer projection length in zig-zag all-trans conformation. $D_\xi = k_B T/\eta_s \xi$ is the diffusion coefficient of the correlation blob.

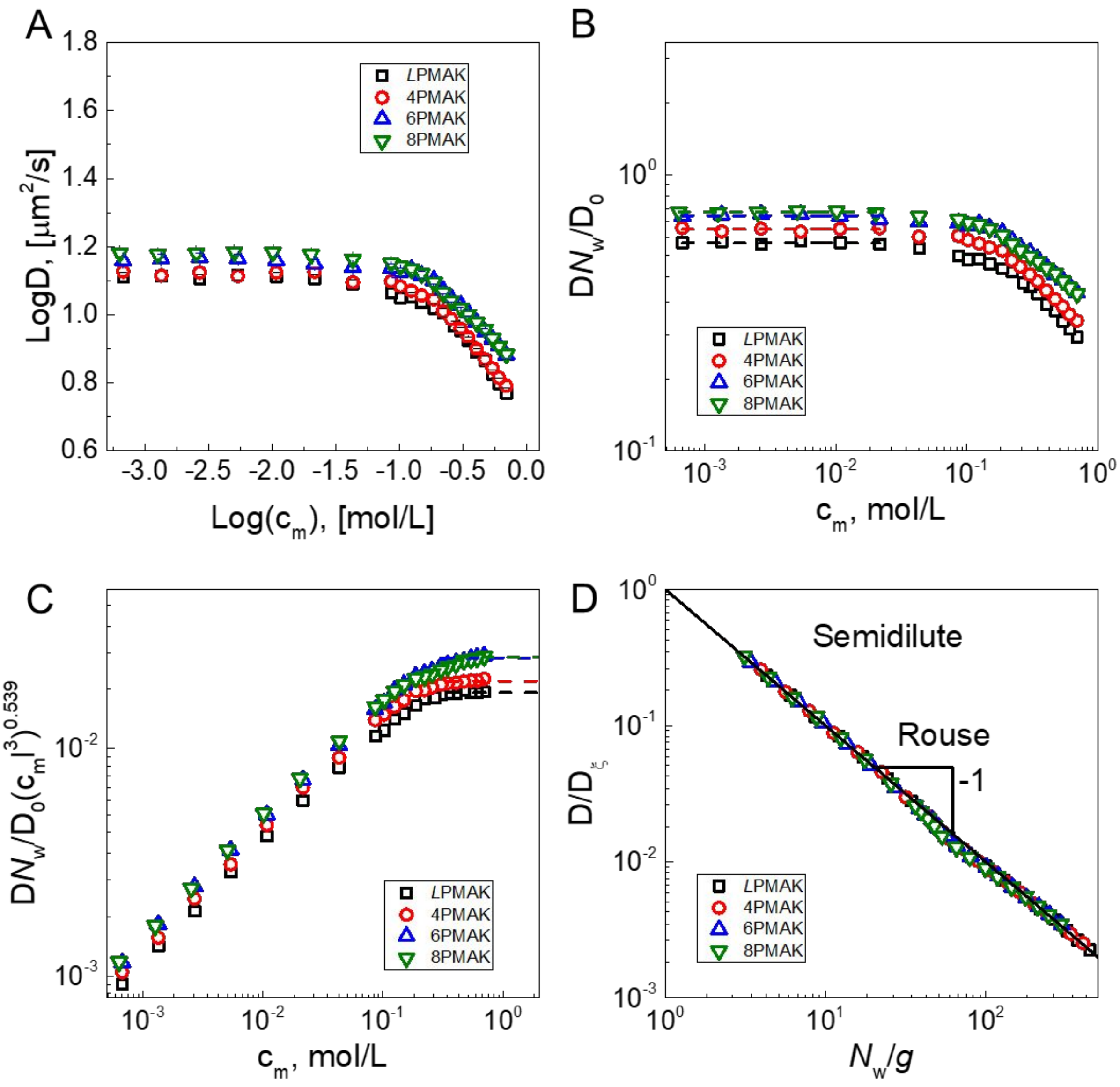

Fig. S9. The dependence of diffusion coefficient D (A) and normalized diffusion coefficient DN_w/D_0 (B) and $DN_w/D_0(c_m l^3)^{0.539}$ (C) on polymer unit concentration in low salt solutions of *L*PMAK (black squares), 4PMAK (red circles), 6PMAK (blue triangles), and 8PMAK (green inverted triangles) in 1*10^{-5} mol/L Tris buffer at pH=9. The dashed lines in (B) correspond to estimated values of B_{pe}/C_ζ equal to 0.561 (black), 0.627 (red), 0.704 (blue), and 0.728 (green). The dashed lines in (C) correspond to estimated values of $C_{p,\mathrm{g}}$ equal to 0.018 (black), 0.020 (red), 0.025 (blue), and 0.025 (green). The normalized diffusion coefficient D/D_ξ as a function of the number of correlation blobs N_w/g (D) with the solid line indicating a scaling dependence in the Rouse regime. $D_0 = k_B T/\eta_s l = 1.59 \times 10^{-8}\ m^2/s$, $T =$ 293.15 K, solvent viscosity $\eta_s = 0.001$ Pa$\cdot$s and $l = 0.255$ nm is the monomer projection length in zig-zag all trans conformation. $D_\xi = k_B T/\eta_s \xi$ is the diffusion coefficient of the correlation blob.

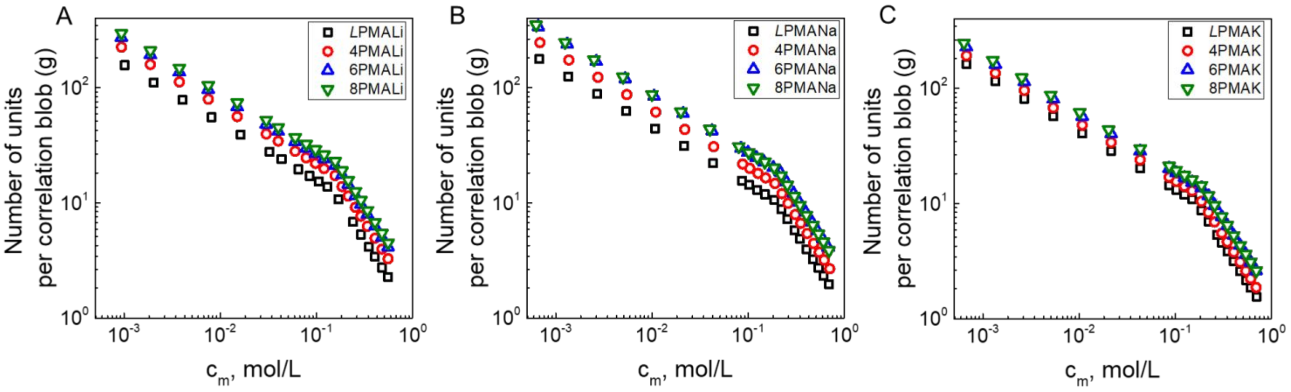

Fig. S10. The effect of polymer concentration on number of polyelectrolyte units per correlation blob g in the low-salt solutions of linear (black squares), 4-arm (red circles), 6-arm (blue triangles), and 8-arm (green inverted triangles) PMALi (A), PMANa (B) and PMAK (C) in $1*10^{-5}$ mol/L Tris buffer at pH=9 and 20 °C.

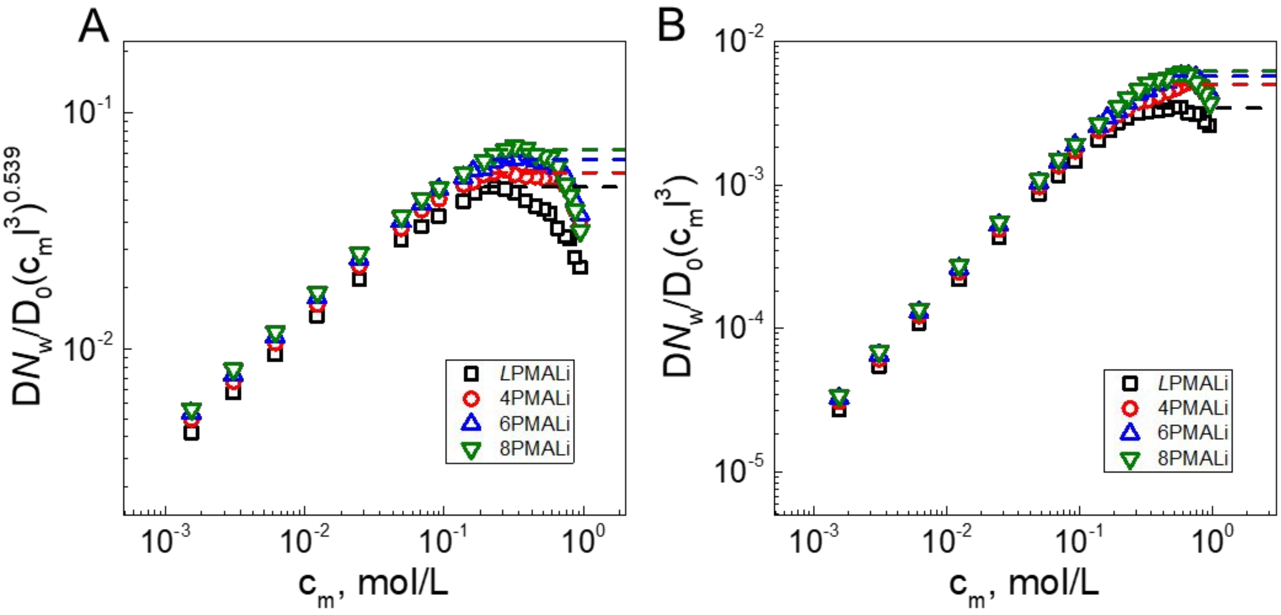

Fig. S11. The log-log dependences of normalized diffusion coefficients $DN_w/D_0(c_m l^3)^{0.539}$ (A) and $DN_w/D_0 c_m l^3$ (B) on polymer unit concentration in aqueous solutions with 1.0 mol/L LiCl of linear PMALi (black squares), 4PMALi (red circles), 6PMALi (blue triangles), and 8PMALi (green inverted triangles) in 1.0 mol/L aqueous solutions of LiCl. The dashed lines in (A) show the estimated values of $C_{p,\mathrm{g}}$ equal to 0.048 (black), 0.056 (red), 0.063 (blue), and 0.068 (green). The dashed lines in (B) show the estimated values of $C_{p,\mathrm{th}}$ equal to 3.41×10^{-3} (black), 4.94×10^{-3} (red), 5.7×10^{-3} (blue), and 6.02×10^{-3} (green). $D_0 = k_B T/\eta_s l$=$1.57 \times 10^{-8} m^2/s$, solvent viscosity $\eta_s = 0.001155 \mathrm{Pa \cdot s}$ for aqueous solutions of 1.0 M LiCl, $T =$ 293.15 K and $l = 0.255\mathrm{nm}$ is the monomer projection length in zig-zag all trans conformation. $D_\xi = k_B T/\eta_s \xi$ is the diffusion coefficient of the correlation blob.

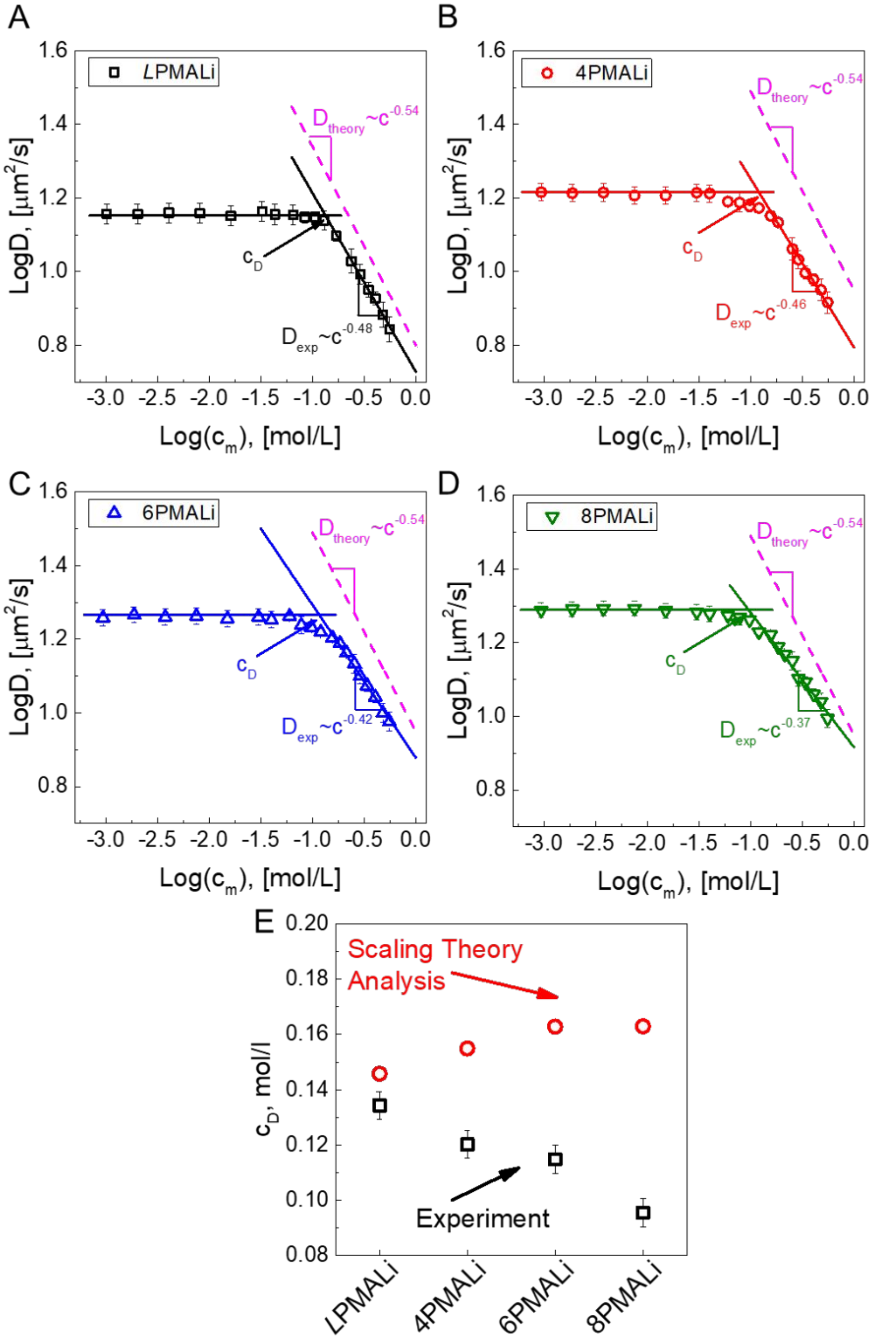

Fig. S12. Concentration dependences of self-diffusion coefficient of *L*PMALi (A), 4PMALi (B), 6PMALi (C), and 8PMALi (D) in 1*10^{-5} mol/L Tris buffer. The solid lines represent the fitting of linear regions of the experimental data (R^2 is 0.995, 0.989, 0.991 and 0.993 for (A), (B), (C) and (D), respectively). The dashed line shows the theoretical dependence of $D \propto c_m^{-0.54}$ predicted by the polyelectrolyte scaling theory. Note that R^2 values were 0.98, 0.96, 0.90 and 0.84 for (A), (B), (C) and (D), respectively, for the data fitted as D ~ $D \propto c_m^{-0.54}$. The intersection of the experimental solid lines gave estimates for overlap electrostatic blob concentration (c_D).

Determination of B-parameters and normalization for the low-salt and high-salt regime

In **eq. 5** in the main text of the paper, the specific values of B-parameters defining conformation of strands as different length scales (see Figure S6) can be determined from the concentration dependence of the self-diffusion coefficient in the low-salt regime by using its normalized value DN/D_0 $(c_m l^3)^{(1-\nu)/(1-3\nu)}$ with the exponent ν corresponding to the different solution regimes, as illustrated in **Table S1**. After rewriting Eq. 5 in the main text leads to the following equation:

$$\frac{B^{2/(3\nu-1)}}{C_\zeta} \approx \frac{DN}{D_0(c_m l^3)^{(1-\nu)/(1-3\nu)}} \qquad \text{(S1)}$$

where B-parameter is equal to B_{pe} , B_g and B_{th} in polyelectrolyte, good solvent and theta solvent regime with exponent ν being equal to 1, 0.588 and 0.5 respectively. Note that for this normalization a numerical coefficient C_ζ and B-parameters cannot be separated since they always enter expression together, thus, additional condition is required to determine their individual values as explained below.

Equation S1 can also be used to extract B-parameters from the experimental data (**Table S2**) in the high-salt regime and replot the self-diffusion data (**Fig. 4D**) similarly to how this was done in the low-salt regime. At this salt concentration, however, we can use an overlap concentration $c_m{}^* l^3 \approx B^3 N_w^{1-3\nu}$ to decouple C_ζ and B-parameter. In the case of stars, we substituted them by equivalent linear chains. The obtained values of C_ζ varied between 4.85 (chains) and 4.44 (8-arms star) (**Table S2**). This allowed us to overlap the data sets for linear chains and stars with the different number of arms in the entire concentration range spanning both dilute and semidilute solutions. For the plot in **Fig. 4D**, we expanded concentration dependence of g into a dilute solution regime, $N/\text{g} << 1$) and set D_ξ/C_ζ equal to the plateau value.

Table S1: Scaling Analysis of Low Salt Aqueous Solutions of PMAA Chains and Stars

Ion	Arch	N_w	B_{pe}/C_ζ	$B_g/C_\zeta^{(3\nu-1)/2}$	c_D [mol/L]
Li^+	Linear	695	0.629	0.218	0.146
Li^+	4 Star	758	0.779	0.240	0.155
Li^+	6 Star	776	0.886	0.254	0.163
Li^+	8 Star	766	0.936	0.260	0.163
Na^+	Linear	695	0.594	0.226	0.194
Na^+	4 Star	758	0.744	0.246	0.193
Na^+	6 Star	776	0.906	0.271	0.213
Na^+	8 Star	766	0.912	0.269	0.201
K^+	Linear	695	0.561	0.214	0.163
K^+	4 Star	758	0.627	0.224	0.166
K^+	6 Star	776	0.704	0.243	0.201
K^+	8 Star	766	0.728	0.244	0.190

B_{pe}/C_ζ is determined from the plateau values of the normalized diffusion coefficient DN_w/D_0, where $D_0 = k_BT/\eta_s$ l= $1.59 \times 10^{-8} m^2/s$, $T =$ 293.15 K, viscosity $\eta_s = 0.001$ Pa · s in aqueous solutions at low-salt concentrations and $l = 0.255$nm is the monomer projection length in zig-zag all-trans conformation. $B_g/C_\zeta^{(3\nu-1)/2} = C_{p,g}^{(3\nu-1)/2}$ is determined from the plateau values of the normalized diffusion coefficient $C_{p,g}$ =$DN_w/D_0(cl^3)^{0.539}$, scaling exponent $\nu = 0.588$, and c_D is the crossover concentration defined as $c_Dl^3 = B_{pe}^3(B_g/B_{pe})^{2/(1-\nu)}$.

Table S2: Scaling Analysis of 1.0M Aqueous LiCl Salt Solutions of PMAA Chains and Stars

Arch	N_w	$C_{p,\mathrm{g}}$	$C_{p,\mathrm{th}}$	B_g	B_th	c^* [mol/L]	c_th [mol/L]	C_ζ	$D(c^*)$ [m^2/s]	g_th	D_th [nm]
Linear	695	4.83×10^{-2}	3.41×10^{-3}	0.575	0.359	0.128	0.318	4.85	3.47×10^{-11}	211	10.33
4 Star	758	5.55×10^{-2}	4.94×10^{-3}	0.597	0.389	0.134	0.524	4.65	3.57×10^{-11}	127	7.39
6 Star	776	6.31×10^{-2}	5.68×10^{-3}	0.622	0.402	0.149	0.541	4.57	3.74×10^{-11}	144	7.62
8 Star	766	6.82×10^{-2}	6.02×10^{-3}	0.634	0.405	0.160	0.519	4.44	3.95×10^{-11}	164	8.07

$C_{p,\mathrm{g}}$ and $C_{p,\mathrm{th}}$ are determined from plateau values of the normalized diffusion coefficient $C_{p,\mathrm{g}} = DN_w/D_0(cl^3)^{0.539}$ and $C_{p,\mathrm{th}} = DN_w/D_0cl^3$, where $D_0 = k_BT/\eta_s l$=1.37 $\times 10^{-8} m^2/s$, $\eta_s = 0.00116\mathrm{Pa\cdot s}$ in aqueous solutions of 1.0 M LiCl, $T =$ 293.15 K and $l = 0.255\mathrm{nm}$ is the monomer projection length in zig-zag all trans conformation. B_g is obtained from the overlap concentrations c^* as $B_\mathrm{g} = \left(c^* l^3/N_w^{(1-3\nu)}\right)^{1/3}$, $l = 0.255\mathrm{nm}$ is the monomer projection length in all trans zig-zag conformation, scaling exponent $\nu = 0.588$, and B_th is obtained from $C_{p,\mathrm{th}}$ as $B_{th} = \left(C_{p,\mathrm{th}} C_\zeta\right)^{1/4}$. Numerical coefficient $C_\zeta = B_\mathrm{g}^{2/(3\nu-1)}/C_{p,\mathrm{g}}$ and $D(c^*)$ is the diffusion coefficient in the dilute solution regime, $c < c^*$. Number of monomers per thermal blob $g_\mathrm{th} = B_{th}^6/(c_{th} l^3)^2$ and its size $D_\mathrm{th} = l\, g_\mathrm{th}^{0.5}/B_\mathrm{th}$.

References

(1) Aliakseyeu, A.; Shah, P. P.; Ankner, J. F.; Sukhishvili, S. A. Salt-Induced Diffusion of Star and Linear Polyelectrolytes within Multilayer Films. *Macromolecules* **2023**, *56*, 5434-5445.

(2) Pristinski, D.; Kozlovskaya, V.; Sukhishvili, S. A. Fluorescence correlation spectroscopy studies of diffusion of a weak polyelectrolyte in aqueous solutions. *The Journal of Chemical Physics* **2004**, *122*, 014907.

(3) LeBel, R. G.; Goring, D. A. I. Density, Viscosity, Refractive Index, and Hygroscopicity of Mixtures of Water and Dimethyl Sulfoxide. *Journal of Chemical & Engineering Data* **1962**, *7*, 100-101.

(4) Rumble, J.: *CRC Handbook of Chemistry and Physics*; CRC Press, 2023.

(5) Dobrynin, A. V.; Jacobs, M. When Do Polyelectrolytes Entangle? *Macromolecules* **2021**, *54*, 1859-1869.

(6) Dobrynin, A. V.; Sayko, R.; Colby, R. H. Viscosity of Polymer Solutions and Molecular Weight Characterization. *ACS Macro Letters* **2023**, *12*, 773-779.
